# Characterization of Diabetic and Non-Diabetic Foot Ulcers Using Single-Cell RNA-Sequencing

**DOI:** 10.3390/mi11090815

**Published:** 2020-08-28

**Authors:** Michael Januszyk, Kellen Chen, Dominic Henn, Deshka S. Foster, Mimi R. Borrelli, Clark A. Bonham, Dharshan Sivaraj, Dhananjay Wagh, Michael T. Longaker, Derrick C. Wan, Geoffrey C. Gurtner

**Affiliations:** 1 Department of Surgery, Division of Plastic and Reconstructive Surgery, Stanford University School of Medicine, Stanford, CA 94305, USA; januszyk@stanford.edu (M.J.); kellenchen@stanford.edu (K.C.); dhenn2@stanford.edu (D.H.); dsfoster@stanford.edu (D.S.F.); mimib@stanford.edu (M.R.B.); cbonham@stanford.edu (C.A.B.); ds311@stanford.edu (D.S.); longaker@stanford.edu (M.T.L.); dwan@stanford.edu (D.C.W.); 2 Stanford Functional Genomics Facility, Stanford University School of Medicine, Stanford, CA 94305, USA; dwagh@stanford.edu

**Keywords:** single cell RNA sequencing, transcriptomics, diabetes, wound healing, tissue repair, fibrosis, cellular ecology

## Abstract

*Background:* Recent advances in high-throughput single-cell sequencing technologies have led to their increasingly widespread adoption for clinical applications. However, challenges associated with tissue viability, cell yield, and delayed time-to-capture have created unique obstacles for data processing. Chronic wounds, in particular, represent some of the most difficult target specimens, due to the significant amount of fibrinous debris, extracellular matrix components, and non-viable cells inherent in tissue routinely obtained from debridement. *Methods:* Here, we examined the feasibility of single cell RNA sequencing (scRNA-seq) analysis to evaluate human chronic wound samples acquired in the clinic, subjected to prolonged cold ischemia time, and processed without FACS sorting. Wound tissue from human diabetic and non-diabetic plantar foot ulcers were evaluated using an optimized 10X Genomics scRNA-seq platform and analyzed using a modified data pipeline designed for low-yield specimens. Cell subtypes were identified informatically and their distributions and transcriptional programs were compared between diabetic and non-diabetic tissue. *Results:* 139,000 diabetic and non-diabetic wound cells were delivered for 10X capture after either 90 or 180 min of cold ischemia time. cDNA library concentrations were 858.7 and 364.7 pg/µL, respectively, prior to sequencing. Among all barcoded fragments, we found that 83.5% successfully aligned to the human transcriptome and 68% met the minimum cell viability threshold. The average mitochondrial mRNA fraction was 8.5% for diabetic cells and 6.6% for non-diabetic cells, correlating with differences in cold ischemia time. A total of 384 individual cells were of sufficient quality for subsequent analyses; from this cell pool, we identified transcriptionally-distinct cell clusters whose gene expression profiles corresponded to fibroblasts, keratinocytes, neutrophils, monocytes, and endothelial cells. Fibroblast subpopulations with differing fibrotic potentials were identified, and their distributions were found to be altered in diabetic vs. non-diabetic cells. *Conclusions:* scRNA-seq of clinical wound samples can be achieved using minor modifications to standard processing protocols and data analysis methods. This simple approach can capture widespread transcriptional differences between diabetic and non-diabetic tissue obtained from matched wound locations.

## 1. Introduction

Diabetes affects nearly 10% of the adult population in the United States, and this number is projected to increase by more than 50% by 2030 [1,2]. Elevated blood glucose, peripheral neuropathy, and associated micro- and macrovascular complications impair wound healing capacity and the ability of diabetic tissue to recover following ischemia. Consequently, nearly half of all diabetic wounds will recur within six months [3]. Chronic, non-healing ulcers and pressure sores in diabetic patients represent the leading cause of non-traumatic limb amputations in the U.S. and contribute to the significant burden of diabetes on healthcare systems worldwide [4,5].

Repair of damaged skin involves the dynamic interplay between resident cells and cells recruited from the circulation [6,7,8,9,10]. Fibroblasts are the principal mesenchymal cell of the dermis and have been extensively studied for their role in modulating collagen and extracellular matrix (ECM) deposition in response to changes in their microenvironment following tissue injury [11,12,13,14]. Myeloid and lymphoid are examples of cell populations derived from hematopoietic precursors which migrate to sites of skin tissue breakdown to assist with wound healing. Although the cells involved in wound healing have largely been identified, the nature of their interactions mediating skin repair and regeneration are complex and remain incompletely understood. Recent studies have highlighted aberrant changes to the cellular ecology in chronic wounds which critically alter healing capacity [15]. To fully elucidate the mechanisms leading to impaired wound healing in diabetes, it is necessary to delineate the cellular and molecular perturbations driving this abnormal tissue state, including changes to the nature and number of critical cell populations such as stem/progenitor cells [15,16].

To date, most studies investigating the cellular milieu of diabetic wounds have used small animal models, with comparatively less work focused on human tissue [17,18]. This is in part due to the numerous challenges encountered with acquiring, storing, and processing human tissue samples. One of the most fundamental challenges that exists in studying human wound healing is that, unlike in mice, human specimens cannot be routinely harvested *en bloc* and are instead collected as medical waste from debridement. This tissue is typically collected in clinics or operating rooms that are remote from laboratories, of low volume, and often stored for prolonged periods at room temperature before subsequent processing.

Ideally, tissue is processed as quickly as possible after harvest in order to preserve cell integrity, viability, and RNA quantity. When immediate processing is not possible, storage on ice can slow down natural degradation (enzymatic or otherwise), and storage within growth serum-supplemented media can nourish cells and preserve viability [19]. However, there is an inherent tradeoff between prolonged time-to-capture and non-physiologic changes to cellular transcriptional signatures. For example, gentler digestion concentrations or longer (slower) centrifuge speeds will reduce agitation of the cells and preserve RNA quality. However, these steps will also increase the total processing time of the cells. Increased time before scRNA-seq capture (both from storage on ice and experimental processing) will increasingly alter the cells’ molecular signatures. Additionally, use of enzymatic digestion solutions optimized for the specific tissue sample type and size can minimize loss of certain (potentially rare) cell populations, such as stem cells. Once cells have been processed into subsequent cellular suspensions for evaluation using single cell-omics platforms, such as the 10X Chromium, the quality of cell capture is influenced by several factors. The principal challenge is achieving the optimal cell concentration to prevent clogging, a risk which is increased when processing cells from sites of injury or in the setting of tumors. Clogging can be minimized by adding DNase or employing a Ficoll step to reduce cellular debris. When clogging occurs during capture, anything captured before the clog can still, fortunately, be sequenced. Clogs that occur early during cellular capture, however, can render the entire sample worthless.

In this work, we demonstrate the feasibility and effectivity of using single-cell RNA-seq to explore the cellular ecology within excised tissue from the wounds of diabetic and non-diabetic patients, maintained on ice within supplemented culture media for prolonged periods (up to 180 min). We describe our methods for processing the clinical samples and demonstrate the effectiveness of capture using minor modifications to standard protocols. Using this approach, we are able to describe differences at the transcriptional level between cells comprising the abnormal foot ulcers of diabetic patients compared to cells from matched plantar foot wounds of non-diabetic patients. We characterize cell populations present within human diabetic and non-diabetic wound tissue, providing a comparative informatic assessment of tissue regeneration and fibrosis that may inform future wound healing studies.

## 2. Materials and Methods

### 2.1. Sample Collection

Wound tissue samples were obtained under an approved IRB (#45287) at the Stanford Advanced Wound Care Clinic (AWCC) by the senior author (GCG). In accordance with Stanford Health Care (SHC) policy, all staff and personnel involved in the study completed HIPAA training and used encrypted computers to store de-identified patient data. The tissue collected in this study would have otherwise been discarded following wound debridement as part of standard-of-care. Thus, this research posed minimal risk to the patients involved, consistent with sound research design outlined by the Stanford University Research Compliance Office. There were no gender or ethnic restrictions for enrollment. A list of HIPAA-compliant subject characteristics is provided in Supplementary Table S1.

### 2.2. Sample Preparation

Debrided diabetic foot ulcer (DFU) tissue was collected from one patient and immediately placed into a solution of Dulbecco’s Modified Eagle Medium (DMEM; Sigma-Aldrich, St. Louis, MO, USA) with 10% fetal bovine serum (FBS; ThermoFisher, Waltham, MA, USA) and stored on normal ice (to keep samples at 4 °C). Then, debrided non-diabetic foot ulcer (NFU) tissue was collected from a second patient in the same fashion 1.5 h later. Samples remained on ice at the wound clinic for 1.5 to 3 h following debridement before transfer to the laboratory for processing. At the laboratory, samples were rinsed three times with 1x phosphate buffered saline (PBS; Fisher Scientific, Waltham, MA, USA) to cleanse samples of media and FBS that would quench the enzymatic digest solutions added next. Samples were cut into ~2–3 mm^3^ pieces with a scalpel and finely minced with fine scissors until a sludge-like consistency (<~0.1 mm^3^ pieces). Although samples were already small prior to mincing (<100 mm^3^), effort was made to ensure that a fine a consistency was reached, as finer tissue pieces have superior cellular yields. The resulting tissue was placed into a 50 mL conical tube containing 20 mL Liberase (Sigma-Aldrich) in PBS at a concentration of 0.5 mg/mL for enzymatic digest. The cell-digest suspensions were constantly agitated (rotated) for a total of 1.5 h at 37 °C. Every half hour, the sample was subjected to maximum speed on a vortex mixer (VWR) for 30 s to physically disrupt any tissue that had clumped together and thus maximize the tissue surface area exposed to enzymatic digestion at all times. The cellular and enzymatic solution was then pipetted through a 100 µm Nylon cell filter (Fisher-Scientific) into a new conical tube, and 20 mL of 10% FBS DMEM was added through the filter to quench the enzymatic reaction and release any cells trapped within the filter, maximizing downstream cell yield. Solutions were then spun at 300× *g* for 8 min at 4 °C in a centrifuge to pellet the cells, resuspended in 20 mL 10% FBS DMEM, and passed through a 70 µm Nylon cell filter. A 20 mL solution of 10% FBS DMEM was added through the filter to collect the remaining cells, and the solution was re-spun to obtain a final cell pellet. This pellet was resuspended to a final cellular concentration of 1200 cells/µL in 0.04% Bovine Serum Albumin (BSA; Sigma-Aldrich) in PBS in accordance with the maximum capture, concentration short of overloading, per specifications from 10X Genomics (Pleasanton, CA, USA).

### 2.3. Single-Cell RNA Sequencing

Single-cell RNA-seq (scRNA-seq) was performed at the Stanford Functional Genomics Facility (SFGF) for droplet-based microfluidic single cell RNA sequencing (scRNA-seq) using the 10x Chromium Single Cell platform (Single Cell 3′ v3, 10X Genomics, Pleasanton, CA, USA). A droplet of the cell suspensions, reverse transcription master mix, and partitioning oil was loaded onto a single cell chip and processed on the Chromium Controller. Reverse Transcription was performed at 53 °C for 45 min. cDNA was amplified for 12 cycles total (BioRad C1000 Touch thermocycler) with cDNA size selected using SpriSelect beads (Beckman Coulter, Indianapolis, IN, USA) and a ratio of SpriSelect reagent volume to sample volume of 0.6. cDNA was analyzed on an Agilent Bioanalyzer High Sensitivity DNA chip for qualitative control purposes. cDNA was fragmented using the proprietary fragmentation enzyme blend for 5 min at 32 °C, followed by end repair and A-tailing at 65 °C for 30 min. cDNA were double-sided size selected using SpriSelect beats. Sequencing adaptors were ligated to the cDNA at 20 °C for 15 min. cDNA was amplified using a sample-specific index oligo as primer, followed by another round of double-sided size selection using SpriSelect beads. Final libraries were analyzed on an Agilent Bioanalyzer High Sensitivity DNA chip for qualitative control purposes. cDNA libraries were sequenced on a HiSeq 4000 Illumina platform aiming for 500,000 reads per cell.

### 2.4. Single Cell RNA-Seq Data Processing, Normalization, and Cell Subpopulation Identification

Base calls were converted to reads using the Cell Ranger (10X Genomics, Pleasanton, CA, USA; version 3.1) implementation *mkfastq* and then aligned against the GRCh38 v3.0.0 (human) genome using Cell Ranger’s count function with SC3Pv3 chemistry and 5,000 expected cells per sample. Cell barcodes representative of quality cells were delineated from barcodes of apoptotic cells or background RNA based on a threshold of having at least 300 unique transcripts profiled, less than 100,000 total transcripts, and less than 10% of their transcriptome of mitochondrial origin. Unique molecular identifiers (UMIs) from each cell barcode were retained for all downstream analysis. Raw UMI counts were normalized with a scale factor of 10,000 UMIs per cell and subsequently natural log transformed with a pseudocount of 1 using the R package Seurat (version 3.1.1) [20]. Aggregated data were then evaluated using uniform manifold approximation and projection (UMAP) analysis over the first 15 principal components [21]. Cell annotations were ascribed using SingleR (version 3.11) against the Human Primary Cell Atlas (HPCA) reference dataset [22]. A recursive analysis of the initial putatively fibroblast subpopulation was performed with SingleR following repartitioning with a resolution parameter of 0.2, from which two of the four subgroups were excluded as keratinocytes and monocytes.

### 2.5. Generation of Characteristic Subpopulation Markers and Enrichment Analysis

Cell-type marker lists were generated with two separate approaches. In the first approach, we employed Seurat’s native *FindMarkers* function with a log fold change threshold of 0.25 using the ROC test to assign predictive power to each gene. However, in order to better account for the mutual information contained within highly correlated predictive genes, we also employed a characteristic direction analysis [23]. The 50 most highly ranked genes from this analysis for each cluster were used to perform gene set enrichment analysis in a programmatic fashion using EnrichR (version 2.1) [24].

### 2.6. Evaluation of Cell Differentiation Status Using CytoTRACE

We utilized the recently developed bioinformatics tool CytoTRACE to compare differentiation states among cells in our dataset [25]. This tool analyzes the number of uniquely expressed genes per cell, as well as other factors like distribution of mRNA content, to calculate a score assessing the differentiation and developmental potential of cells. This analysis was performed using default parameters for each cell in our dataset.

### 2.7. Evaluation of Wound Fibroblasts in Pseudotime

We utilized the Monocle 3 tool in order to assign pseudotime values to the cells within our dataset [26]. Monocle 3 examines transcriptional similarities between all cells within a given embedding and orders them such that cells more similar to each other are closer in “pseudotime”, while those more transcriptionally distinct are further apart. By comparing pseudotime values, we can follow the transcriptional progression among cell states in our dataset.

## 3. Results

### 3.1. Data Processing and Quality Control

**Figure 1:**
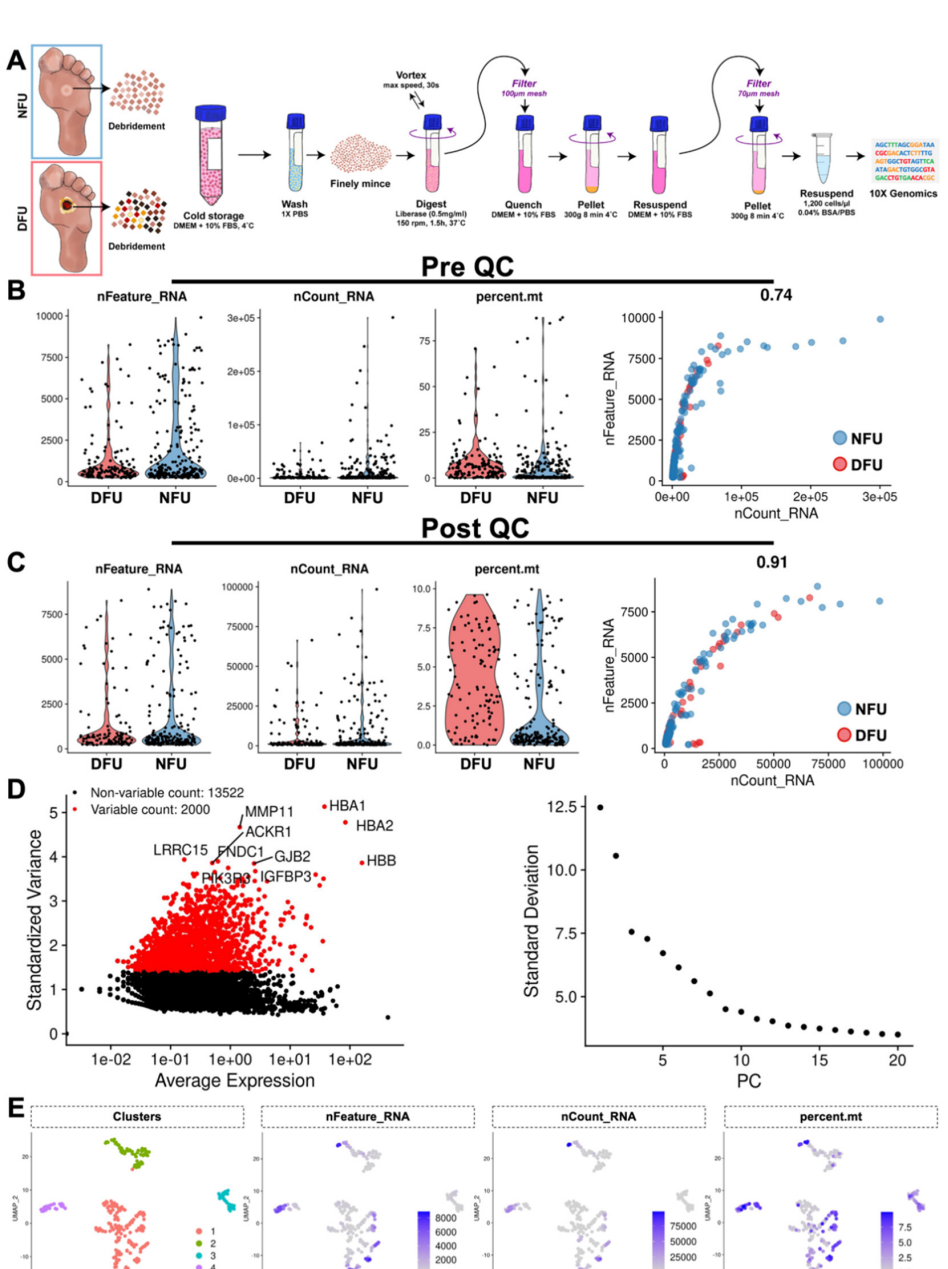
Data processing and quality control. (**A**) Samples were collected from the wound clinic and processed into cellular suspensions for single cell RNA sequencing. (**B**) Each sequenced cell is plotted to show their number of RNA features (nFeature_RNA), absolute numerical count of RNA (nCount_RNA), and percent mitochondria (percent.mt). (**C**) Post quality control (QC) filtering of these cells. (**D**) Highly variable genes within this dataset are used to partition cells along 15 principal components (PC). (**E**) Four transcriptionally distinct subpopulations are identified, with feature plots of nFeature_RNA, nCount_RNA, and percent.mt overlaid.

Following tissue harvest, samples spent either 3 h (diabetic) or 1.5 h (non-diabetic) in FBS media on ice prior to extraction into a single cell suspension (Figure 1A). Cell counting performed at this time identified 95,000 diabetic cells and 44,000 non-diabetic cells. Given the comparatively low number of cells, we were able to sequence to depths of greater than 500,000 reads per cell for both diabetic and non-diabetic tissue. After sequencing, we successfully aligned 83.6% of diabetic and 83.4% of non-diabetic barcoded sequence fragments to the human transcriptome (GRCh38-3.0.0). This resulted in 141 and 243 candidate cells, respectively, that met minimum thresholds of 200 unique genes. These cells were then evaluated for their fraction of mitochondrial RNA, an early sign of cell membrane compromise (Figure 1B). Interestingly, we observed elevated mitochondrial RNA in the diabetic cells compared to their non-diabetic counterparts (8.5% vs. 6.6%, *p* = 0.14), which may be attributable in part to increased cold ischemia time. Final thresholds for cell viability were set at 10% mitochondrial RNA, at least 300 unique genes, and fewer than 100,000 total gene counts. This reduced the number of cells to 108 (diabetic) and 199 (non-diabetic), which were then used for the remainder of our analysis (Figure 1C). Following log-normalization, highly variable genes (HVGs) were identified within our dataset and used to partition the cells along 15 principal components (Figure 1D). We performed an initial blinded Louvain-based cluster analysis and identified four transcriptionally distinct subpopulations, each of which was well-represented by cells from both samples and did not appear skewed according to our basic quality-control metrics (Figure 1E) [27].

### 3.2. Identification of Characteristic Wound Healing Cell Subtypes from Unsorted Populations

**Figure 2:**
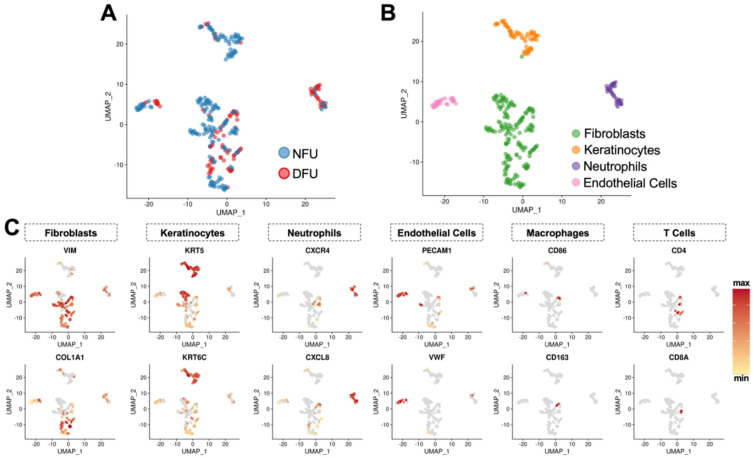
Identification of characteristic wound healing cell subtypes from unsorted populations. (**A**) UMAP embedding of cells clustered independently of experimental group, labeled as either non-diabetic foot ulcer (NFU) or diabetic foot ulcer (DFU). (**B**) UMAP embedding of cells labeled by cell type using the Human Primary Cell Atlas (HPCA) reference dataset and automated annotation tool SingleR. (**C**) Feature plots of cell subtype specific marker genes.

**Figure 3:**
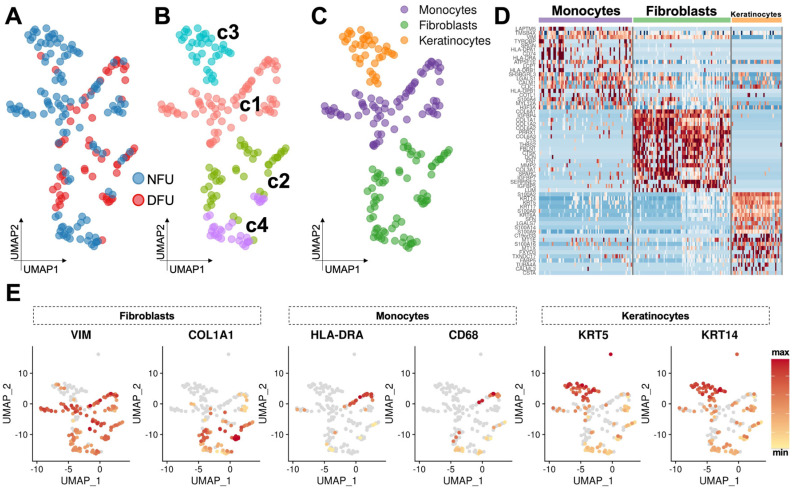
Re-clustering of initial fibroblast clusters demonstrate four provisional subclusters. (**A**,**B**) Recursive re-clustering reveals four provisional subclusters (*c1*–*c4*) which further identify as (**C**) fibroblasts, monocytes, or keratinocytes. (**D**) Heatmap demonstrating top differential genes between these newly refined cell annotations. (**E**) Manual evaluation of key cell type markers.

To further characterize these cell groups, we employed the automated annotation tool SingleR. [22]. Comparing cells against the Human Primary Cell Atlas (HPCA) reference dataset, we obtained congruence scores for each cluster against candidate cell subtypes from this database (Table S2). As expected, we observed fibroblasts, keratinocytes, endothelial cells, and immune cells (Figure 2A,B). However, our fibroblast cluster received a significantly lower confidence score compared to the other three clusters, suggesting that these data may have been under-clustered using default parameters. This was further supported by evaluation of cell subtype specific marker genes, which showed that these cells exhibited patchwork characteristics of several known wound cell types (Figure 2C). We therefore re-clustered this data subset in a recursive fashion using a lower resolution threshold and identified four provisional subclusters (*c1*–*c4*) with significantly distinct transcriptional profiles (Figure 3A,B). Two of these (*c2* and *c4*) appeared to be true fibroblast populations, whereas the others were monocytes (*c1*) and a second population of keratinocytes (*c3*) (Figure 3C) and were excluded from subsequent fibroblast analysis. Manual evaluation of key cell type markers further supported these automated annotations (Figure 3D,E).

### 3.3. Diabetic Wound Fibroblasts Exhibit Increased Expression of Pro-Fibrotic and Inflammatory Markers and Decreased Expression of Anti-Apoptotic Markers

**Figure 4:**
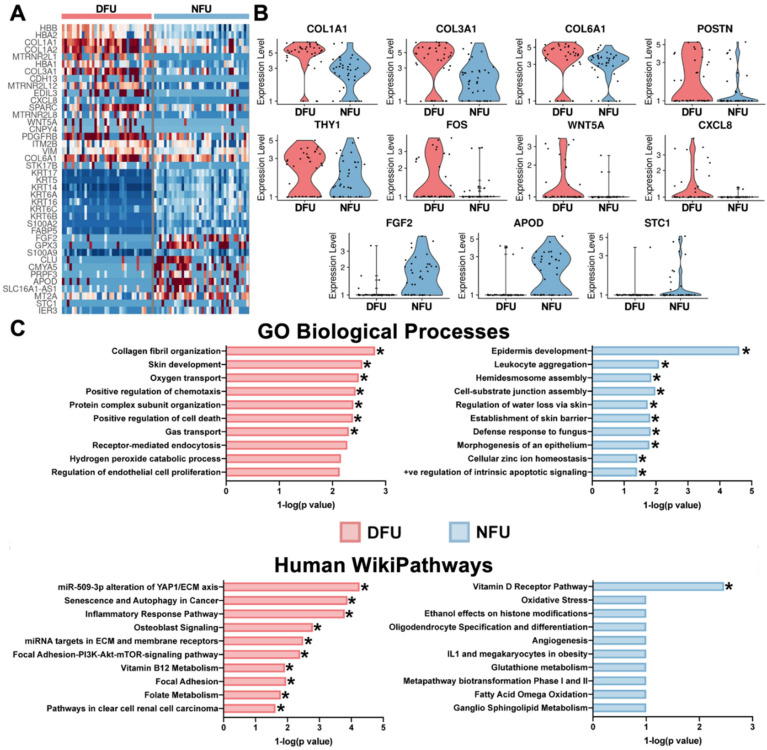
Diabetic wound fibroblasts exhibit increased expression of known pro-fibrotic markers. (**A**) Heatmap of differential genes between DFU and NFU in the refined fibroblasts. (**B**) Violin plots of specific differential genes. (**C**) Enrichment pathway analysis using the top twenty-five genes for each group. * (*p* < 0.05).

Using this refined definition of fibroblasts, we then examined the expression patterns of notable genes in wound healing and diabetes biology [15,17,28]. In aggregate, we found that diabetic wound fibroblasts exhibited increased expression of *COL1A1*, *COL3A1*, and *COL6A1*, associated with ECM deposition and fibrosis [29]; *FOS*, *POSTN*, *THY1*, *WNT5A*, and *ACTA2*, which are widely established pro-fibrotic and myofibroblast markers [30]; and *CXCL8*, *MXD1,* and *CD44*, indicative of an elevated inflammatory state (Figure 4A,B, Supplementary Figure S1) [31,32,33,34,35]. Compared to their non-diabetic counterparts, diabetic cells exhibited lower expression of *FGF2*, associated with fibroblast mitogenic and cell survival activities [36]; *APOD*, associated with fibroblast regeneration [37]; and *CSTB*, *SMARCA4*, and *HSPA9*, which have all been shown to inhibit apoptosis [38,39,40]. Enrichment analysis of genes up-regulated in diabetic cells demonstrated increases in pathways related to the YAP1/ECM axis, inflammatory response, ECM membrane receptors, and Focal Adhesion-PI3K-Akt-mTOR-signaling. Diabetic transcriptional programs also showed enrichment for skin development and collagen fibril organization, with decreases in epidermis development and establishment of skin barriers (Figure 4C).

### 3.4. Diabetes Alters the Distribution of Fibroblast Subpopulations

**Figure 5:**
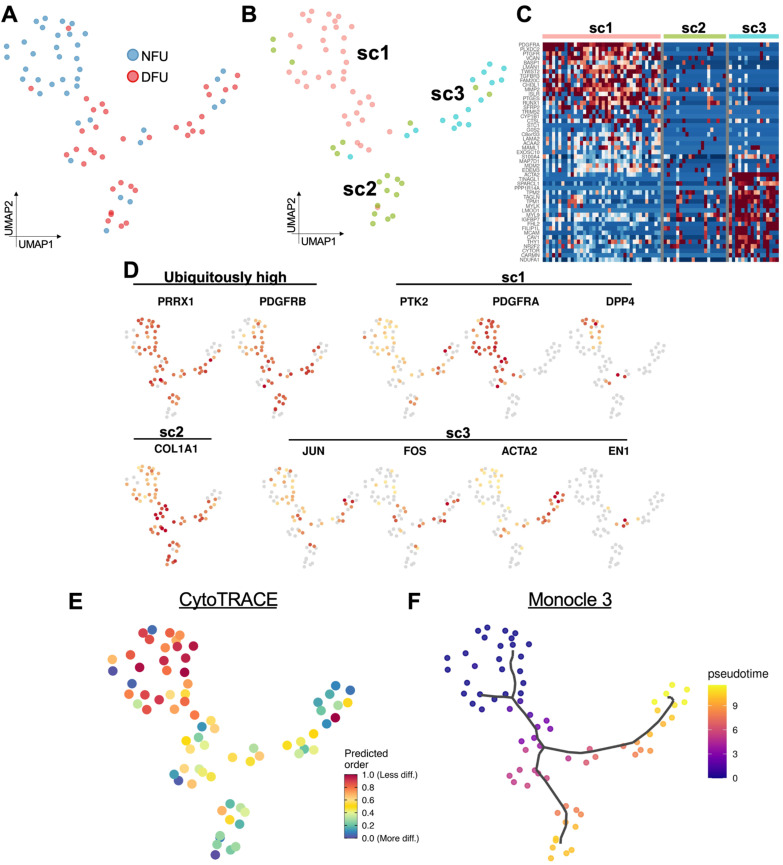
Diabetes alters the distribution of fibroblast subpopulations. (**A**) UMAP of refined fibroblasts by experimental group. (**B**) Re-clustering of these cells reveals three subclusters (*sc1*–*sc3*). (**C**) Heatmap of differential genes between the three subclusters. (**D**) Feature plots of genes show fibroblast subpopulations previously characterized by our laboratory and others. (**E**) CytoTRACE analysis shows differentiation potential of these cells. (**F**) Monocle 3 pseudotime analysis shows diverging transcriptional trajectories of wound fibroblasts.

Our laboratory and others have previously characterized fibroblast subpopulations in murine wound healing [11,41,42]. Here, we evaluated whether our human wound samples exhibited similar expression patterns to those murine populations. In particular, we examined fibroblasts based on their expression of the genes *EN1*, *PRRX1*, *DPP4*, *JUN*, *FOS*, *COL1A1*, *PDGFRA*/*B*, *YAP1*/*PTK2*, and *ACTA2*. We performed a re-clustering of cells based solely on expression of these markers and identified three transcriptionally-distinct fibroblast subpopulations (Figure 5A–C). The first, designated as *subcluster 1* (*sc1*), corresponded to cells with differentially elevated expression of *PTK2*, *PDGFRA*, and *DPP4* (Figure 5B–D). These likely represent the traditionally-defined PDGFRA+ fibroblasts, and the increase in *PTK2* (FAK) expression supports the mechano-sensing properties of these cells, which our group has robustly demonstrated as a critical driver of dermal fibrosis [43,44]. The second subcluster, *sc2*, appears to show low expression of most pro-fibrotic markers, but all maintained large quantities of *COL1A1* transcripts. These cells may represent a basal population of collagen producing fibroblasts, maintaining the generation of extracellular matrix elements in this chronic non-healing state. The final group, *subcluster 3*, demonstrated differential activation of *JUN*, *FOS*, and *ACTA2*. These cells likely represent a human analog to the pro-fibrotic subpopulation of cells that has been previously described by our group and others [45]. Furthermore, these data suggest that a small subpopulation of cells highly expressing *EN1* may specifically drive the transcriptional shift into this pro-fibrotic state.

Interestingly, diabetic and non-diabetic tissue showed considerable differences in their representation among these three clusters. Non-diabetic foot ulcer (NFU) cells were distributed primarily within *sc1*, the traditional fibroblast subpopulation, while diabetic foot ulcer (DFU) cells were comparatively enriched among the collagen producing *sc2* and pro-fibrotic *sc3* populations. Additional analysis with CytoTRACE suggested that DFU fibroblasts were in aggregate more differentiated than non-diabetic wound cells, potentially a consequence of the global metabolic changes associated with diabetes that may drive fibroblast differentiation into the more pathologic *sc2* and *sc3* cell states (Figure 5E). Indeed, when we set the root of differentiation origin at the NFU cells in *sc1* using Monocle3, we can also see how these fibroblasts progress along transcriptional trajectories starting at *sc1* and then branching into either *sc2* or *sc3* with increasing pseudotime (Figure 5F).

## 4. Discussion

Human wound tissue is difficult to study on a single cell level due to multiple factors, including sample scarcity and low tissue viability. Furthermore, delays in the transfer of tissue, from time of harvest in the clinic or operating room until laboratory processing, pose significant risks to cellular integrity. scRNA-seq analysis of cells that contribute to wound healing is also inherently challenging due to the significantly lower cell-to-debris ratio compared to intact (uninjured) tissue or to pathologically proliferative tissue (e.g., tumors). Furthermore, compared to animal models, collecting wound tissue in humans occurs under much less well-controlled conditions, often in the context of surgical procedures. Tissue collected for research from patients during debridement is also generally extremely limited in quantity to prevent any adverse side effects to the patient, further reducing the ability to obtain good cell yields. Due to all of these factors, effective scRNA-seq wound protocols have yet to be developed. While several studies have presented scRNA-seq data concerning human skin at homeostasis [46,47], the study of fibroblasts and other cell types from human chronic wounds has yet to be robustly demonstrated.

In this study, we leverage our clinical wound healing expertise and understanding of the fundamentals of scRNA-seq technology to provide the first detailed application of scRNA-seq in human diabetic and non-diabetic wound tissue. We provide key quality control metrics and demonstrate that our approach can successfully capture sufficient numbers of high-quality cells to identify characteristic wound subpopulations. We illustrate how these cell subtypes can be distinguished in silico based on gene expression profiles without the need to perform FACS, which is costly, labor-intensive, and associated with increased time-to-capture, increased physical manipulation, and inherent bias based on marker selection. This allowed us to successfully compare the transcriptional programs of canonical wound cell types in diabetic and non-diabetic samples, as well as identify differences in the relative proportions of putative subpopulations associated with diabetes.

Single cell analysis has revolutionized the ability to explore complex tissues, leading to major advances in our understanding of cellular biology, molecular physiology, and translational medicine. Early efforts to characterize single cell gene expression led to the development of microfluidic-based chips to achieve massively parallel qPCR assays; however, these were limited in the number of genes (typically 48 or 96) and cells (typically 48 or 96) that could be simultaneously evaluated [48,49]. Even as recently as 2015, most single cell analysis was done with the Fluidigm C1 platform, which relied on 48 × 48 or 96 × 96 well microfluidics chips to deliver very small and precise volumes (single cells) into isolated chambers for cDNA amplification. Recent advancements in the ability to randomly capture and manipulate individual cells in nanoliter droplet emulsion has eliminated the 96 well plate limitation and allowed for significantly improved automated cell capture. These droplet-based technologies combine a flow of cellular suspension within reagents with timed addition of oil at set intervals to create immiscible fluid droplets [50]. In combination with recent advances in technology for the untargeted amplification of entire single cell transcriptomes, this automated cell isolation approach now permits the capture and sequencing of complete single cell transcriptional programs with the throughput necessary for the characterization of complex tissue such as healing wounds [50,51].

Even with these technological advances, single cell analysis of human wound tissue presents significant challenges, and we devoted considerable effort to optimizing our approach to generate the highest quality single cell suspensions from clinical samples. Cells isolated from injury or tumor specimens in particular are predisposed to clumping and adhere together excessively, generally requiring multiple cell filtering steps. Diabetic cells may clump even more due to increased cell adhesion molecules, further exacerbating this issue for diabetic wounds [52,53]. Other considerations include the use of a red blood cell lysis step or reagents to decrease debris, each of which require additional washing and centrifugation which further extend the length of time before cell capture, with commensurate tradeoffs in cellular quality and decreases in physiologic transcriptional levels. All of these decisions are framed in the context of avoiding plating cells to expand cell numbers, since this is known to significantly alter gene expression [54].

The final, and perhaps most important, consideration is the decision of whether or not to employ cell sorting. At a minimum, MACS or FACS can be beneficial to isolate live cells and potentially enrich for a population of interest (for example, a mouse wound fibroblast population can be enriched by lineage gating against CD45, CD326, CD324, CD31, Ter119, and Tie2) [55]. However, these techniques can severely traumatize cells, particularly those coming from an injury specimen that may be stressed and have high metabolic demand at baseline. Additional drawbacks to FACS include significant decreases in cell yield, increased time between cell isolation and droplet capture, introduction of potential contaminants, and again potential biased enrichment for certain populations over others. For droplet capture, a manual cell counting step is generally still required after FACS as the sorted number of cells counted from FACS machines are not sufficiently accurate. MACS is quicker and does not require a dedicated flow cytometry core or specialized training, but purification is much lower [56]. Although FACS may be required for the examination of extremely rare and well-defined populations of interest, in this study we chose to avoid cell sorting and demonstrated that cells could still be categorized and purified in silico, through differences in their transcriptomes, in a fashion that permitted downstream informatic analysis of their alterations in diabetes.

Cellular ecology, developmental trajectories, and dynamics can now be understood with dramatically increased granularity due to the advent of high-throughput-omics technologies [57]. However, challenges requiring careful optimization remain, particularly for primary human tissue specimens. Here, we demonstrate a clinically relevant protocol that allows us to process debrided tissue in order to compare the transcriptional programs underlying chronic wound states in the context of diabetic and non-diabetic cellular ecology. Although the present study was not powered to draw broad conclusions about diabetic wound biology, we believe that our findings are of high translational importance as we have, for the first time, successfully interrogated human tissue samples derived from diabetic and non-diabetic wounds using single cell RNA sequencing. Our approach allowed us to identify transcriptionally-distinct subpopulations of human fibroblasts, which have yet to be examined in this context. These results can serve as comparators for scRNA-seq data obtained from mouse samples as well as those obtained from other fibrotic conditions in humans. Identifying shared gene expression pathways affecting tissue healing is a critical step towards the development of novel therapies. As diabetes is a major healthcare burden and its incidence is rising worldwide, it is of critical importance to develop novel and effective treatment strategies for diabetic tissue injury. Fibroblasts are the major cell type of the dermis, of vital importance for normal physiologic wound healing, and as such were the focus of our analysis. Our findings may facilitate the development of novel therapeutics aiming at correcting pathologic subpopulation changes of fibroblasts related to diabetes. As personalized medicine is gaining importance, future clinical treatment algorithms for diabetic patients might incorporate scRNA-seq of debrided wound tissue from the clinic, as analyzed in our study, in order to identify the specific transcriptional signature of fibroblast subpopulations of individual patient wounds. This may allow future clinicians to tailor their therapeutics to the individual patient’s cellular wound ecology and achieve superior outcomes.

## Supplementary Materials

The following information can be found at https://www.mdpi.com/2072-666X/11/9/815/s1. Supplemental Table S1, Supplemental Table S2, and Supplemental Figure S1 are available online as Supplementary Materials.
