# Supplementary material for: Characterization of Diabetic and Non-Diabetic Foot Ulcers Using Single-Cell RNA-Sequencing
Source: Micromachines (Basel). 2020 Aug 28;11(9):815. doi: 10.3390/mi11090815 (PMC7570277; doi:10.3390/mi11090815)
Supplement: Supplementary file 1 [file micromachines-11-00815-s001.pdf]

Supplementary Table 1. Subject demographics.

	**Diabetic Foot Ulcer**	**Plantar (Non-Diabetic) Pressure Ulcer**
**Gender**	Male	Male
**Age**	55	90
**Cold Ischemia Time (in 10% FBS media)**	1.5 hrs	3.0 hrs

Supplementary Table 2. Congruence scores for each cluster against candidate cell subtypes using the automated annotation tool SingleR using the Human Primary Cell Atlas (HPCA) reference dataset.

Cell Type	Cluster			
	Cluster1	Cluster2	Cluster3	Cluster4
Neutrophils	0.2637539	0.1778974	0.6873177	0.2255986
Monocytes	0.4072305	0.279386	0.5663235	0.3210987
MEP	0.334222	0.3256397	0.2019464	0.3054729
CD4+ T-cells	0.322949	0.275375	0.2557965	0.2963024
Tregs	0.3132264	0.2746157	0.2682454	0.2767004
CD4+ Tcm	0.3180245	0.2785369	0.2677064	0.281688
CD4+ Tem	0.3269443	0.2839054	0.2712913	0.2824864
CD8+ Tcm	0.3280422	0.2807525	0.2726947	0.2804991
CD8+ Tem	0.3272097	0.2706733	0.2741275	0.2773866
NK cells	0.3269174	0.2646455	0.3150644	0.2792845
naive B-cells	0.307616	0.2581024	0.2986889	0.2977399
Memory B-cells	0.3056279	0.2667161	0.2891223	0.2906208
Class-switched memory B-cells	0.3113318	0.2697008	0.2928975	0.2898009
HSC	0.3496716	0.295291	0.2370775	0.3376862
MPP	0.3475003	0.3164852	0.2516936	0.3415848
CLP	0.3500479	0.2848172	0.2373085	0.2971385
GMP	0.3643887	0.3136537	0.2630997	0.3216182
Macrophages	0.437638	0.3506188	0.4322862	0.3344808
CD8+ T-cells	0.3227887	0.2796308	0.3356223	0.2736523
Erythrocytes	0.3630036	0.3500648	0.2232735	0.323497
Megakaryocytes	0.3450738	0.3341088	0.2204974	0.344902
CMP	0.3424134	0.3156131	0.4341795	0.3162707
Macrophages M1	0.4419945	0.3573396	0.373684	0.3272991
Macrophages M2	0.4166397	0.3708212	0.329898	0.3518724
Endothelial cells	0.4843405	0.4521842	0.13313	0.6772455
DC	0.4348255	0.3301969	0.3810592	0.3398959
Eosinophils	0.2557496	0.2037673	0.4881594	0.2429877
Plasma cells	0.3094286	0.3187595	0.237726	0.3069675
Chondrocytes	0.6221791	0.4674089	0.1142751	0.4839637
Fibroblasts	0.6201043	0.4843506	0.1091398	0.5092274
Smooth muscle	0.6004802	0.4674223	0.1014581	0.4974195
Epithelial cells	0.489848	0.7177673	0.1244614	0.4917009
Melanocytes	0.4751381	0.4652431	0.1325311	0.4396141
Skeletal muscle	0.5128924	0.4043033	0.1150099	0.4977156
Keratinocytes	0.4579401	0.7080667	0.1196829	0.4287909
mv Endothelial cells	0.4552655	0.4548601	0.1355012	0.6734385
Myocytes	0.5752	0.4591048	0.0948635	0.4793548
Adipocytes	0.6242797	0.4092869	0.2783241	0.5713446
Neurons	0.3207616	0.3244942	0.0068876	0.2778464
Pericytes	0.58121	0.4755354	0.1473425	0.4687521
Preadipocytes	0.5860866	0.4633824	0.0986187	0.4570288
Astrocytes	0.523914	0.4666439	0.0570388	0.4407191
Mesangial cells	0.5077231	0.5376044	0.0906405	0.4744043

Supplemental Figure 1

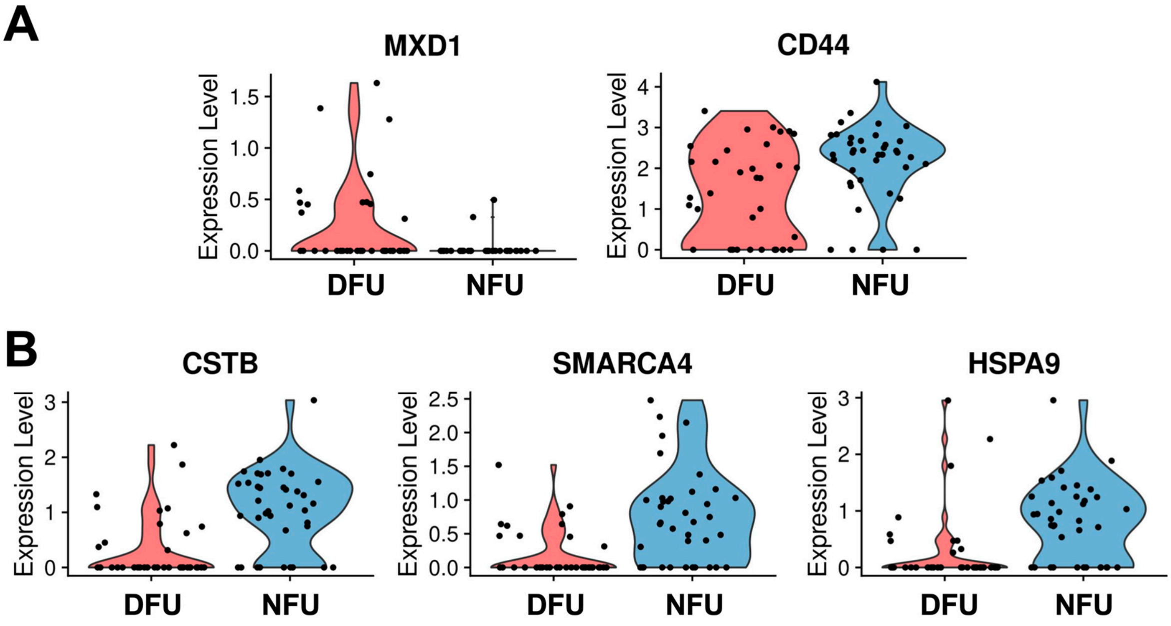

Supplemental Figure 1. Diabetic wound fibroblasts exhibit increased expression of inflammatory and decreased expression of anti-apoptotic markers. Violin plots of differentially expressed inflammatory (**A**) and anti-apoptotic (**B**) genes.
